# Dynamic compression counteracts IL-1β induced inducible nitric oxide synthase and cyclo-oxygenase-2 expression in chondrocyte/agarose constructs

**DOI:** 10.1186/ar2389

**Published:** 2008-03-18

**Authors:** TT Chowdhury, S Arghandawi, J Brand, OO Akanji, DL Bader, DM Salter, DA Lee

**Affiliations:** 1 School of Engineering and Materials Science, Queen Mary, University of London, Mile End Road, London, E1 4NS, UK; 2 Queens Medical Research Institute, 47 Little France Cresent, Edinburgh University, EH16 4TJ. UK

## Abstract

**Background:**

Nitric oxide and prostaglandin E_2 _(PGE_2_play pivotal roles in both the pathogenesis of osteoarthritis and catabolic processes in articular cartilage. These mediators are influenced by both IL-1β and mechanical loading, and involve alterations in the inducible nitric oxide synthase (iNOS) and cyclo-oxygenase (COX)-2 enzymes. To identify the specific interactions that are activated by both types of stimuli, we examined the effects of dynamic compression on levels of expression of iNOS and COX-2 and involvement of the p38 mitogen-activated protein kinase (MAPK) pathway.

**Methods:**

Chondrocyte/agarose constructs were cultured under free-swelling conditions with or without IL-1β and/or SB203580 (inhibitor of p38 MAPK) for up to 48 hours. Using a fully characterized bioreactor system, constructs were subjected to dynamic compression for 6, 12 and 48 hours under similar treatments. The activation or inhibition of p38 MAPK by IL-1β and/or SB203580 was analyzed by western blotting. iNOS, COX-2, aggrecan and collagen type II signals were assessed utilizing real-time quantitative PCR coupled with molecular beacons. Release of nitrite and PGE_2 _was quantified using biochemical assays. Two-way analysis of variance and the *post hoc *Bonferroni-corrected *t*-test were used to examine data.

**Results:**

IL-1β activated the phosphorylation of p38 MAPK and this effect was abolished by SB203580. IL-1β induced a transient increase in iNOS expression and stimulated the production of nitrite release. Stimulation by either dynamic compression or SB203580 in isolation reduced the IL-1β induced iNOS expression and nitrite production. However, co-stimulation with both dynamic compression and SB203580 inhibited the expression levels of iNOS and production of nitrite induced by the cytokine. IL-1β induced a transient increase in COX-2 expression and stimulated the cumulative production of PGE_2 _release. These effects were inhibited by dynamic compression or SB203580. Co-stimulation with both dynamic compression and SB203580 restored cytokine-induced inhibition of aggrecan expression. This is in contrast to collagen type II, in which we observed no response with the cytokine and/or SB203580.

**Conclusion:**

These data suggest that dynamic compression directly influences the expression levels of iNOS and COX-2. These molecules are current targets for pharmacological intervention, raising the possibility for integrated pharmacological and biophysical therapies for the treatment of cartilage joint disorders.

## Introduction

The mechanical environment is an important factor that maintains articular cartilage in a healthy state. Mechanical signals generated under normal physiological loading conditions will activate mechanotransduction pathways and drive biochemical events that regulate chondrocyte function and activity [1-4]. It is well established that proinflammatory cytokines such as IL-1β act as the key mediators of cartilage breakdown and stimulate the release of nitric oxide (NO) and prostaglandin (PG)E_2_, via induction of inducible isoforms of the nitric oxide synthase (iNOS) and cyclo-oxygenase (COX)-2 enzymes [5-9]. There is growing evidence that mechanical stimulation inhibits the release of NO and PGE_2 _by articular chondrocytes [10-18]. Thus, mechanical strain acts in an anti-inflammatory manner that may influence the progression of osteoarthritis (OA). However, the molecular mechanisms that underlie specific mechanotransduction pathways are complex and vary depending on the type of mechanical stimuli and pathological environment of the tissue.

The fundamental pathways that play a role in increasing the release of NO and PGE_2 _by IL-1β involve activation of members of the mitogen-activated protein kinase (MAPK) pathway, namely extracellular signal-regulated kinase (ERK)-1/2, p38 and c-Jun amino-terminal kinase (JNK) families, and the transcription factors activator protein-1 and nuclear factor-κB (NF-κB) [19-27]. These studies demonstrated strong stimulation of p38 MAPK by IL-1β and the subsequent induction of iNOS and COX-2 expression in articular chondrocytes. Thus, the potential of p38 MAPK as drug target in cartilage disease has led to the development of several inhibitors by pharmaceutical companies. However, no study has examined the involvement of the p38 MAPK pathway in response to both IL-1β and mechanical loading.

Mechanical stimulation in the form of static or intermittent compression of varying loading modalities, including shear stress or tension, may influence the signal transduction pathways activated by IL-1β [28-37]. For instance, loading studies that apply physiological levels of compression to chondrocytes have demonstrated a role for the integrins in mediating the mechanotransduction process, including downregulation of NO and PGE_2 _release, both in the presence and absence of IL-1β, utilizing iNOS and COX-2 specific inhibitors [16-18,38,39]. The nature of the mechanical loading regimen and model system will therefore determine whether mechanical signals will prevent or induce these inflammatory mediators. Accordingly, the present study examines the interplay between mechano-sensitive and cytokine-sensitive pathways, and determines the effects of IL-1β and dynamic compression on the expression levels of iNOS and COX-2 and involvement of the p38 MAPK pathway.

## Materials and methods

### Isolation of chondrocytes and culture in agarose constructs

This study involves bovine cells procured from a local abbatoir with authorization from the relevant meat inspectors (Dawn Cardington, Bedfordshire, UK). It does not involve humans, human tissue, or experimentation on animals. Full-depth slices of articular cartilage were dissected from the proximal surface of the metacarpalphalangeal joints of 18-month-old cattle [40,41] and diced. The tissue was then incubated on rollers for 1 hour at 37°C in Dulbecco's modified Eagle's medium (DMEM) supplemented with 20% (vol/vol) foetal calf serum plus 2 μmol/l l-glutamine, 5 μg/ml penicillin, 5 μg/ml streptomycin, 20 mmol/l Hepes buffer and 0.85 μmol/l l-ascorbic acid, plus 700 units/ml pronase. It was subsequently incubated for a further 16 hours at 37°C in medium supplemented with 100 units/ml collagenase type XI (All Sigma Chemical Co., Poole, UK). The chondrocyte suspension was washed and cell viability assessed using trypan blue. Chondrocytes were finally re-suspended in media at a cell concentration of 8 × 10^6 ^cells/ml. The cell suspension was added to an equal volume of molten 6% (weight/vol) agarose type VII (Sigma-Aldrich, Poole, UK) in Earle's Balanced Salt Solution (Sigma Chemical Co., Poole, UK) to yield a final cell concentration of 4 × 10^6 ^cells/ml in 3% (weight/vol) agarose. The cell/agarose suspension was transferred into a sterile stainless steel mould, containing holes 5 mm in diameter and 5 mm in height and allowed to gel at 4°C for 20 minutes to yield cylindrical constructs. Chondrocyte/agarose constructs were equilibrated in culture in 1 ml DMEM plus 1 × ITS liquid media supplement (Sigma-Aldrich) at 37°C in 5% carbon dioxide for 24 hours.

### Temporal effects of IL-1β under free-swelling conditions

Constructs were cultured in 1 ml DMEM + 1 × ITS supplemented with 0 or 10 ng/ml IL-1β (Peprotech EC Ltd, London, UK) and/or 10 μmol/l SB203580 (4-[4-fluorophenyl]-2-[4-methylsulfinylphenyl]-5-[4-pyridyl]1H-imidazole; a selective inhibitor of p38 MAPK) [24,42] under free-swelling conditions for 0, 0.75, 1.5, 3, 6, 12, 24, or 48 hours (Merck Chemicals, Nottingham, UK). At the specified time points, representative constructs were snap frozen in liquid nitrogen and stored at -80°C before extraction of mRNA. The corresponding media were stored at -20°C before biochemical analysis.

### Application of dynamic compression

A fully characterised bioreactor system (Zwick Testing Machines Ltd, Leominster, UK) was used to apply physiological levels of dynamic compressive strain to chondrocyte/agarose constructs, as detailed previously [38-41]. Equilibrated constructs were transferred into individual wells of a 24-well culture plate (Costar, High Wycombe, UK) and mounted within the bioreactor apparatus. One millilitre of DMEM plus 1 × ITS was introduced into each well. The media were supplemented with 0 or 10 ng/ml IL-1β and/or 10 μmol/l SB203580. Control constructs were unstrained but were maintained within the bioreactor system. Strained constructs were subjected to a dynamic compressive strain ranging from 0% to 15% in a sinusoidal waveform at a frequency of 1 Hz, as previously described [38-41]. The constructs were cultured at 37°C/5% carbon dioxide for 6, 12, or 48 hours. At the end of each experiment, the constructs were snap frozen in liquid nitrogen and stored at -80°C before extraction of mRNA. The corresponding media were stored at -20°C before biochemical analysis.

### Protein extraction and analysis by Western blotting

Following IL-1β stimulation, chondrocyte/agarose constructs (n = 3) were washed with ice-cold phosphate-buffered saline containing 100 μmol/l Na_3_VO_4 _and pulverized in ice-cold lysis buffer containing 1% Igepal, 1 mmol/l Na_3_VO_4 _(both from Sigma Aldrich) and protease inhibitor cocktail (Roche Diagostics, Lewes, UK). The cell/agarose lysis solution was left on ice for 30 minutes and centrifuged at 13,000 rpm for 15 minutes. Supernatants were collected and protein concentration was determined by Folin-Lowry method on the Dynatech MR5000 microplate reader (Dynatech, Alexandria, VA, USA). Equal amounts of protein (400 μg) were separated by 12% SDS-PAGE and transferred to polyvinylidene fluoride membranes (Millipore Immobilon-P; Sigma Aldrich). Membranes were blocked in buffer containing 1 × Tris-buffered saline (TBS) plus 0.1% Tween-20, and 1% nonfat milk for 2 hours at room temperature, and then washed five times with TBS plus 0.1% Tween-20.

Membranes were incubated with a polyclonal rabbit antibody for phospho-p38 MAPK (Thr180/Tyr182) at a dilution of 1:1,000 (New England Biolabs Ltd, Hitchin, UK). After washing extensively with TBS plus 0.1% Tween-20, membranes were incubated with horseradish peroxidase-linked secondary antibody for 1 hour and the binding was detected using Enhanced Chemiluminescence Plus Western blotting detection system, in accordance with the manufacturer's instructions (Amersham, Buckinghampshire, UK). Membranes were stripped with a solution containing 62.5 mmol/l Tris (pH 6.8), 2% SDS and 100 mmol/l β-mercaptoethanol for 30 minutes at 50°C, and then reprobed using a phosphorylation state-independent antibody for p38 MAPK and α-tubulin, which served as an internal control (Cell Signalling).

### RNA extraction, cDNA synthesis and real-time PCR

Total RNA was isolated from individual constructs using protocols described in the QIAquick^® ^Spin gel extraction and Rneasy^® ^kits (Qiagen, West Sussex, UK), as previously described [43]. Following the manufacturer's instructions, Ambion's DNA-*free *DNase treatment and removal reagents were used to eliminate any contaminating DNA from the RNA sample (Ambion Applied Biosystems, Warrington, UK). RNA was quantified using the Nanodrop ND-1000 spectrophotometer (LabTech, East Sussex, UK) and stored in 40 μl RNase-free water at -80°C until reverse transcription could be performed using manufacturer's protocols from the Stratascript™ First-Strand cDNA synthesis kit (Stratagene, Amsterdam, The Netherlands). Briefly, 200 ng of total RNA was reverse transcribed in a 20 μl reaction volume using the manufacturer-supplied oligo(dT) primers. Minus reverse transcriptase (NoRT) control reactions were prepared for each sample by omitting the Stratascript™ reverse transcriptase.

Real-time quantitative PCR assays coupled with molecular beacons were performed in 25 μl reaction mixtures containing 1 μl cDNA, 12.5 μl Brilliant^® ^QRT-PCR Master Mix, primer pairs and probes listed in Table 1, and nuclease free PCR grade water to 25 μl. Each sample was run in duplicate using the 96-well thermal system of the MX3000P QPCR instrument (Stratagene). Thermocycling conditions comprised an initial polymerase activation step at 95°C for 10 minutes, followed by 35 cycles at 95°C for 30 seconds, at 55°C for 1 minute and at 72°C for 1 minute. In order to screen for contamination of reagents or false amplification, PCR controls were prepared for each sample by preparing identical reaction mixtures except for the addition of the no template control (NTC). NoRT (minus reverse transcriptase) controls were additionally included in each PCR assay.

**Table 1:** Description of the Beacon designer sequences used to quantify gene expression and real-time reaction efficiencies of PCR assays

Gene	Accession number	Sequences	Product size (base pairs)	Efficiency
iNOS	U14640	Probe: 5'-FAM-CGCGATCCCTGCTTGGTGGCGAAGATGAGCGATCGCG-DABCYL-3' Forward: 5'-GTAACAAAGGAGATAGAAACAACAGG-3' Reverse: 5'-CAGCTCCGGGCGTCAAAG-3'	81	1.98 ± 0.06
COX-2	AF031698	Probe: 5'-FAM-CGCGATCGTCAGAAATTCGGGTGTGGTACAGTTGATCGCG-DABCYL-3' Forward: 5'-CGAGGTGTATGTATGAGTGTAGG-3' Reverse: 5'-GTTGGGAGTGGGTTTCAGG-3'	82	1.99 ± 0.03
Aggrecan	U76615	Probe: 5'-FAM-CGCGATCCACTCAGCGAGTTGTCAGGTTCTGAGATCGCG-DABCYL-3' Forward: 5'-TGGTGTTTGTGACTCTGAGG-3' Reverse: 5'-GATGAAGTAGCAGGGGATGG-3'	79	1.97 ± 0.05
Collagen type II	X02420	Probe: 5'-FAM-CGCGATGCGTCAGGTCAGGTCAGCCATATCGCG-DABCYL-3' Forward: 5'-AAACCCGAACCCAGAACC-3' Reverse: 5'-AAGTCCGAACTGTGAGAGG-3'	70	2.00 ± 0.05
GAPDH	U85042	Probe: 5'-HEX-CGCGATCCACCATCTTCCAGGAGCGAGATCCGATCGCG-DABCYL-3' Forward: 5'-TTCAACGGCACAGTCAAGG-3' Reverse: 5'-TTCAACGGCACAGTCAAGG-3'	75	2.03 ± 0.01

The Beacon Designer software was used to design forward and reverse primer and probe sequences for molecular beacon applications and were synthesized by Sigma Genosys Ltd, Cambridge, UK. Secondary structures were avoided using the Mfold programme and sequences were analyzed using Basic Local Alignment Search Tool to verify specificity. Probes contain fluorescein (FAM) or 6-carboxyhexafluorescein (HEX) as a 5'-reporter dye and 4-(4'-dimethylaminophenylazo)benzoic acid (DABCYL) as 3'-quencher. Note that the arm sequences are underlined. COX, cyclo-oxygenase; GAPDH, glyceraldehyde 3-phosphate dehydrogenase; iNOS, inducible nitric oxide synthase.

### Molecular beacon design and characterization for real-time PCR

Molecular beacons were introduced for real-time detection of PCR products and were synthesized from oligonucleotides (Sigma Genosys Limited, Cambridge, UK) using the Beacon Designer software (Premier Biosoft International, California, USA). Probes have a hairpin structure and contain fluorescein (FAM) or 6-carboxyhexafluorescein (HEX) as the 5'-reporter dye and 4-(4'-dimethylaminophenylazo)benzoic acid as the 3'-quencher (Table 1). Sequences were designed to avoid regions of cross homology and analyzed using the Basic Local Alignment Search Tool to verify specificity. Additionally, templates were folded and secondary structures avoided using MFold programme and beacon hairpin melting temperatures were calculated using the Zucker software. Primer accession numbers (EMBL [European Molecular Biology Laboratory]; Table 1) were obtained from previously published studies utilizing bovine chondrocytes [44-46]. Primers used in PCR experiments with molecular beacons produced amplicons, which were between 70 and 82 base pairs (Table 1).

PCR efficiencies for optimal primer pair and probe concentrations were derived from standard curves (n = 3) by preparing a 10-fold serial dilution of cDNA from a sample that represented the untreated control at time zero conditions. The real-time PCR efficiencies (E) of amplification for each target was defined according to the following relationship: E = 10^(-1/slope)^. The R^2 ^value of the standard curve exceeded 0.9998 and revealed efficiency values presented in table 1.

### Data normalization and statistical analyses

Fluorescence data was collected during the annealing stage of amplification and data was analyzed using the MxPro™ QPCR software (version 3.0; Stratagene). Baselines and thresholds were automatically set by the software and used after manual inspection. The cycle threshold (C_t_) value for each duplicate reaction was expressed as the mean value and the results were exported as tab-delimited text files into Microsoft Excel for further analysis. The data obtained by PCR assay for glyceraldehyde 3-phosphate dehydrogenase (GAPDH) was validated as a reference gene by displaying the C_t _values as box and whisker plots, and the distribution examined under free swelling and mechanical loading conditions (data not shown). The C_t _values for GAPDH remained stable with no changes detected under all culture conditions, suggesting its suitability as a reference gene.

Relative quantification of iNOS, COX-2, aggrecan and collagen type II signals was accomplished by normalizing each target gene to GAPDH and to the calibrator sample by a comparative C_t _approach [47,48]. For the free-swelling experiments, the difference in cycle threshold (ΔC_t_) for the target was calculated by subtracting the mean C_t _value for the time zero control (calibrator) from the C_t _value of the target sample. For the mechanical loading studies, ΔC_t _was calculated by subtracting the mean C_t _value for the unstrained, no treatment control (calibrator) from the target sample. The ΔC_t _of the target was then normalized to the ΔC_t _of the reference gene, namely GAPDH. Thus, for each sample, the ratio of the relative expression level of target ΔCt and reference ΔCt was calculated, as shown in the following equation.

Ratio of the relative  exp⁡ression level=(1+ETarg⁡et)Targ⁡etΔCt (Mean calibrator−sample)(1+ERe⁡ference)Re⁡ferenceΔCt (Mean calibrator−sample)

E represents the efficiencies obtained for the target and reference gene. ΔCt_target _represents the difference in C_t _values for the mean calibrator or sample for the target gene. ΔC_tReference_represents the difference in C_t _values for the mean calibrator or sample for the reference gene GAPDH. Ratios were expressed on a logarithmic scale (arbitrary units).

### Nitrite and prostaglandin E2 analysis

Quantification of nitrite and PGE_2 _release were previously described in detail [16-18,38]. Absolute concentrations of nitrite (μmol/l), a stable end-product of NO, were determined in the media using a spectrophotometric method based on the Griess reaction. PGE_2 _production was measured in the culture media using an enzyme immunoassay kit (Amersham Biosciences, Buckinghamshire, UK).

### Statistical analysis

For the time course studies under free-swelling conditions, data represent the mean and standard error of the mean values of six replicates from three separate experiments. Two-way analysis of variance and the *post hoc *Bonferroni-corrected *t*-test was used to examine data for constructs cultured in the presence or absence of IL-1β and/or SB203580. For the mechanical loading experiments, data represent the mean and standard error of the mean values of replicates indicated in the individual figure legend. Statistical analysis was performed by a two-way analysis of variance and the *post hoc *Bonferroni-corrected *t*-tests to compare differences between unstrained and strained constructs cultured under the different treatment conditions. Data were also examined between unstrained constructs cultured in the absence and presence of the cytokine and/or inhibitor. In all cases, a level of 5% was considered statistically significant (*P *< 0.05).

## Results

### IL-1β activates the phosphorylation of p38 MAPK

**Figure 1:**
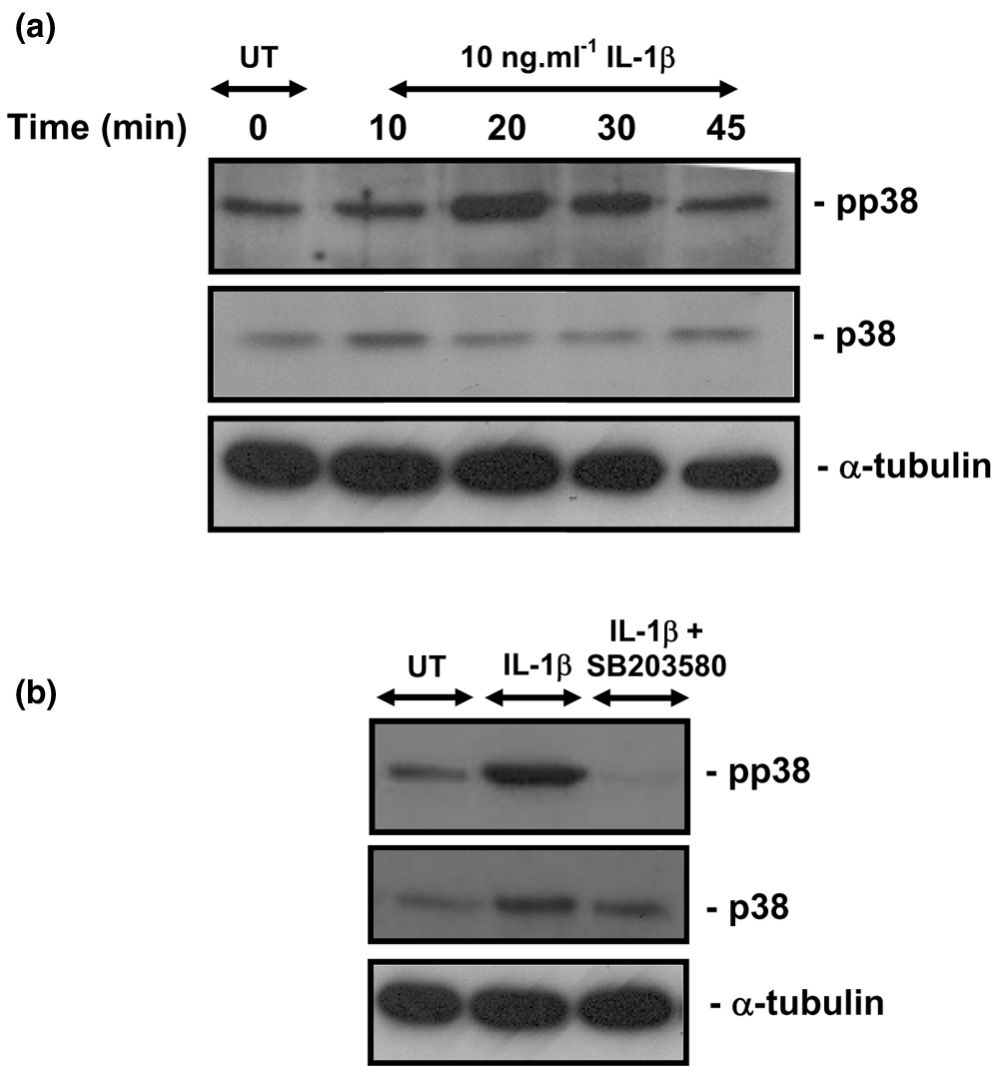
**Activation of p38 MAPK by IL-1β**. p38 phosphorylation by IL-1β in chondrocyte/agarose constructs cultured under free-swelling conditions **(a) **in the presence or absence of 10 ng/ml IL-1β for up to 45 minutes or **(b) **with IL-1β and 10 μmol/l SB203580 for 20 minutes. Phospho-p38 MAPK was analyzed for each test condition using a phosphorylation state specific anti-p38 (Thr180/Tyr182) antibody (upper panel), against total p38 MAPK (middle panel) and α-tubulin as a loading control (lower panel). All cell extracts were subjected to Western blot analysis. Each band corresponds to three constructs pooled from two separate experiments. MAPK, mitogen-activated protein kinase; UT, untreated.

We examined the ability of IL-1β to activate the p38 pathway by Western blot analysis using antibodies directed against the Thr180/Tyr182 phosphorylated p38 (Figure 1). The activation of p38 MAPK was detected 20 minutes after the addition of IL-1β and declined thereafter, when compared with time zero. Co-stimulation with IL-1β and the p38 MAPK inhibitor (10 μmol/l SB203580) abolished the activation of p38 at 20 minutes in chondrocyte/agarose constructs (Figure 1b).

### IL-1β induced iNOS and COX-2 and inhibited aggrecan expression

**Figure 2:**
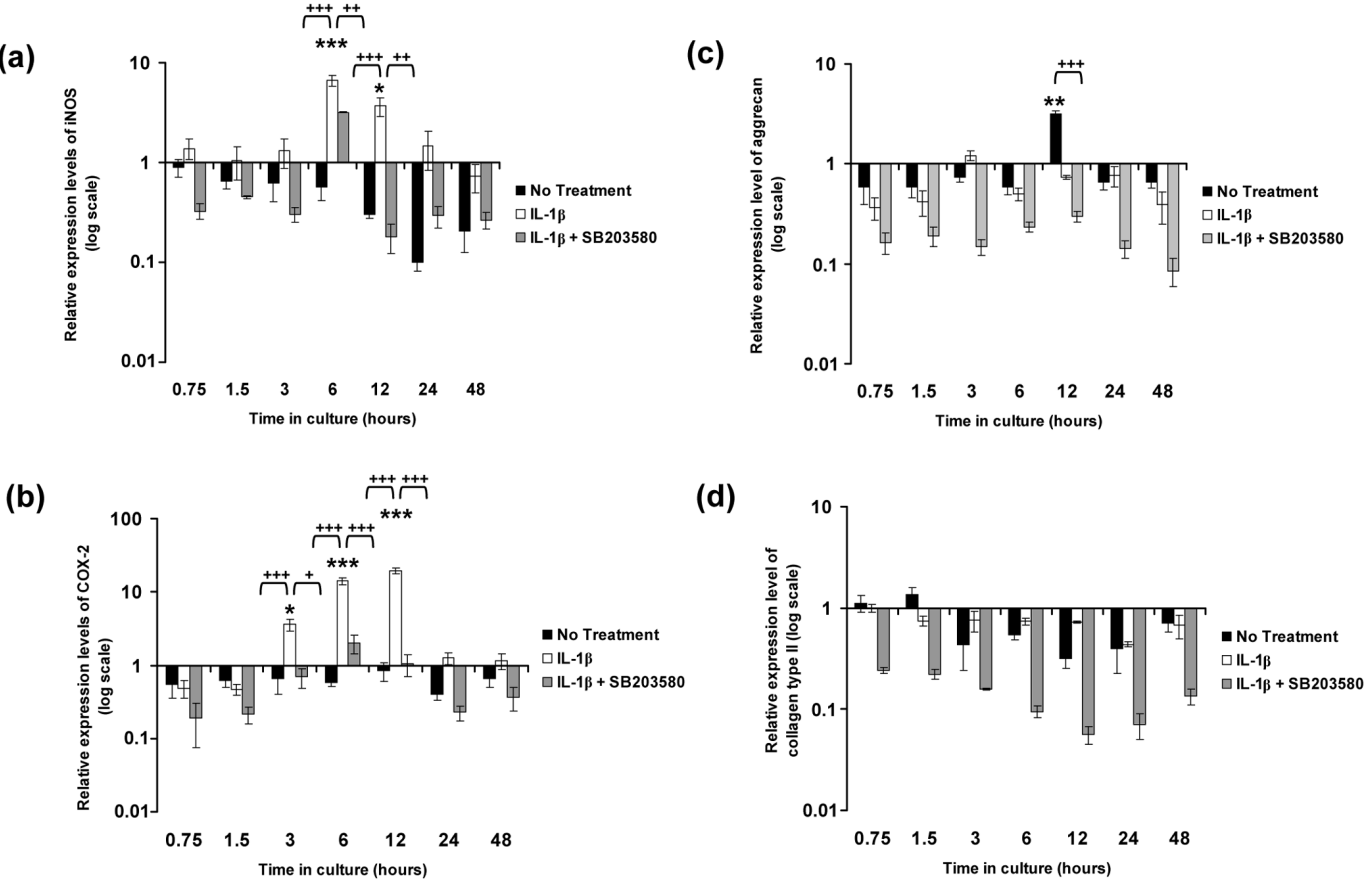
**IL-1β stimulates iNOS and COX-2 expression**. Temporal profile of IL-1β on **(a) **iNOS, **(b) **COX-2, **(c) **aggrecan and **(d) **collagen type II expression by chondrocyte/agarose constructs cultured under free-swelling conditions with 0 or 10 ng/ml IL-1β and/or 10 μmol/l SB203580. Bars represent the mean and standard error of the mean of six replicates from three separate experiments. The ratio of the relative expression level for the target gene was calibrated to the mean value at time = 0 and normalized to the reference gene GAPDH. Ratios were expressed on a logarithmic scale (arbitrary units). Two-way analysis of variance with *post hoc *Bonferroni-corrected *t*-tests was used to compare data under the different treatments: *P < 0.05, **P < 0.01 and ***P < 0.001 for comparisons between time zero with IL-1β; ^+^*P *< 0.05, ^++^*P *< 0.01 and ^+++^*P *< 0.001 for comparisons between untreated with IL-1β or IL-1β with IL-1β plus SB203580. COX, cyclo-oxygenase; GAPDH, glyceraldehyde 3-phosphate dehydrogenase; iNOS, inducible isoforms of the nitric oxide synthase.

Figure 2 illustrates the effects of IL-1β on the relative expression levels of iNOS, COX-2, aggrecan and collagen type II in constructs cultured under free-swelling conditions in the presence and absence of the p38 MAPK inhibitor SB203580. In the absence of the cytokine, the expression levels of iNOS and COX-2 appeared to decrease over a period of 48 hours when compared with time zero (Figure 2 panels a and b, respectively). However, the level of downregulation was not statistically significant when compared with time zero. IL-1β induced a transient increase in iNOS levels, with a peak in expression levels at 6 hours (sevenfold increase; *P *< 0.001) and 12 hours (fourfold increase; *P *< 0.05), which decreased thereafter (Figure 2a). At 6 hours, SB203580 partially reduced the IL-1β induced expression of iNOS to approximately fourfold when compared with constructs treated with IL-1β only (*P *< 0.01; Figure 2a). The IL-1β induced iNOS expression was downregulated with the p38 MAPK inhibitor at 12 hours (*P *< 0.01; Figure 2a). The induction of COX-2 by IL-1β was detected at 3 hours (fourfold increase; *P *< 0.05) and 6 hours (14-fold increase; *P *< 0.001), with a peak in expression at 12 hours (19-fold increase; *P *< 0.001) when compared with time zero (Figure 2b). The presence of SB203580 inhibited the IL-1β induced expression of COX-2 (*P *< 0.05 at 3 hours; *P *< 0.001 at 6 and 12 hours; Figure 2b).

In the absence of the cytokine, there was a downregulation of aggrecan expression up to 6 hours of culture when compared with time zero (Figure 2c). Aggrecan expression levels peaked at 12 hours (threefold increase; *P *< 0.01; Figure 2c) when compared with time zero and was not significantly detected at any other time point. At 12 hours, aggrecan expression was inhibited by the presence of the cytokine (*P *< 0.001) but was not significantly influenced by the addition of SB203580 (Figure 2c). In contrast, the expression levels of collagen type II appeared to be consistently downregulated in the presence or absence of the cytokine or with the addition of the inhibitor (Figure 2d). However, the level of inhibition under the various treatment conditions did not differ significantly relative to that at time zero.

### IL-1β stimulates the production of nitrite and PGE_2 _release

**Figure 3:**
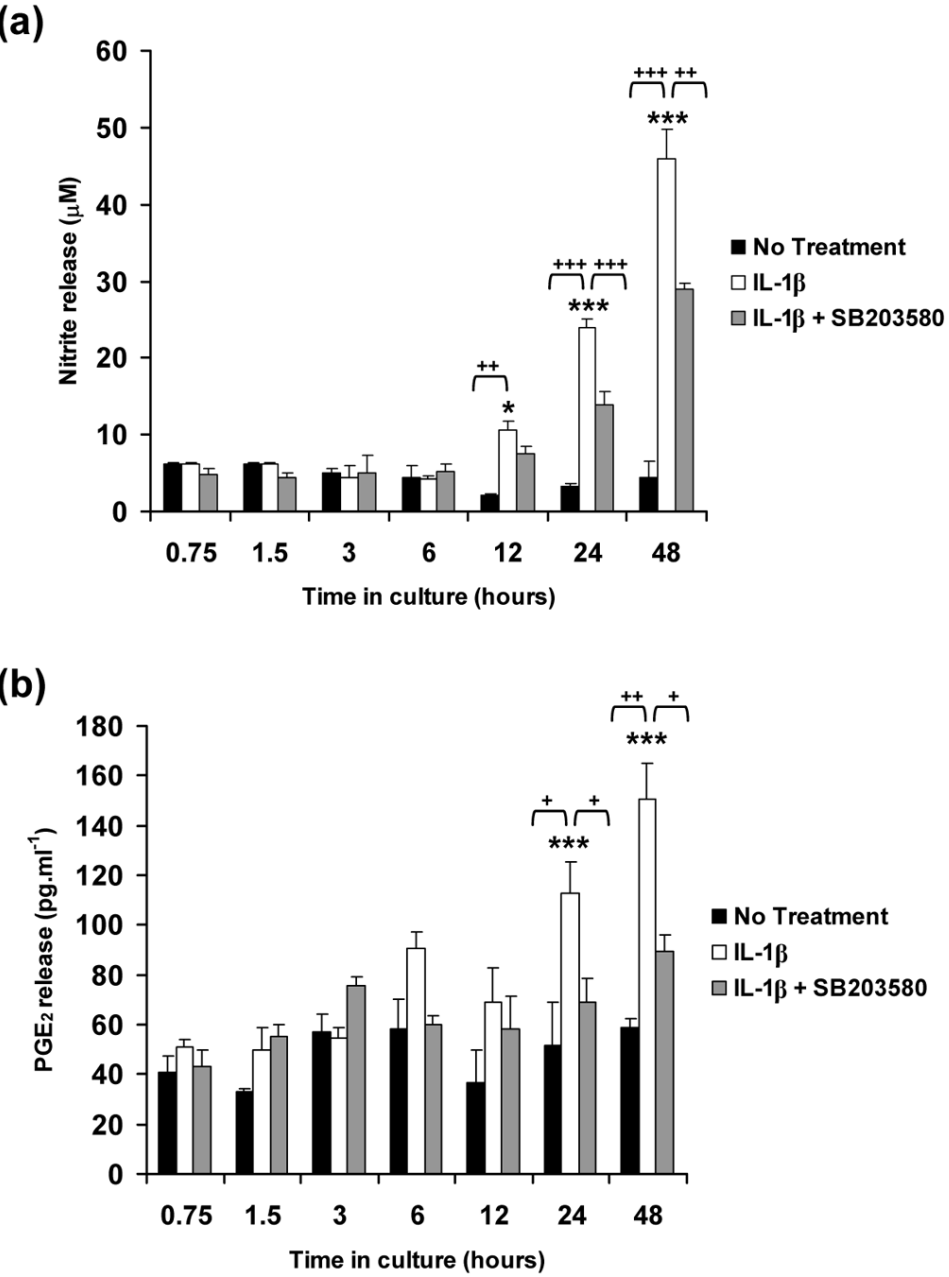
**IL-1β stimulates the production of nitrite and PGE_2 _release**. Temporal profile of IL-1β on the production of **(a) **nitrite and **(b) **PGE_2 _release by chondrocyte/agarose constructs cultured under free-swelling conditions with 0 or 10 ng/ml IL-1β and/or 10 μmol/l SB203580. Bars represent the mean and standard error of the mean of six replicates from three separate experiments. Two-way analysis of variance with *post hoc *Bonferroni-corrected *t*-tests was used to compare data under the different treatments: *P < 0.05 and ***P < 0.001 for comparisons between time zero with IL-1β; ^+^*P *< 0.05, ^++^*P *< 0.01 and ^+++^*P *< 0.001 for comparisons between untreated with IL-1β or IL-1β with IL-1β plus SB203580. PG, prostaglandin.

The cumulative production of nitrite and PGE_2 _release were assessed as presented in Figure 3. In the absence of the cytokine, the levels of nitrite and PGE_2 _release did not change over the 48-hour culture period. IL-1β induced nitrite and PGE_2 _release with significant differences measured at 24 and 48 hours (all *P *< 0.001; Figure 3 panels a and b, respectively). SB203580 partially reduced the IL-1β induced nitrite release at 24 hours (24 μmol/l decrease to 14 μmol/l) and at 48 hours (46 μmol/l decrease to 29 μmol/l). SB203580 abolished the IL-1β induced PGE_2 _release to basal levels (both *P *< 0.05; Figure 3b).

### Dynamic compression inhibited IL-1β induced iNOS and COX-2 expression, production of nitrite and PGE_2 _release

**Figure 4:**
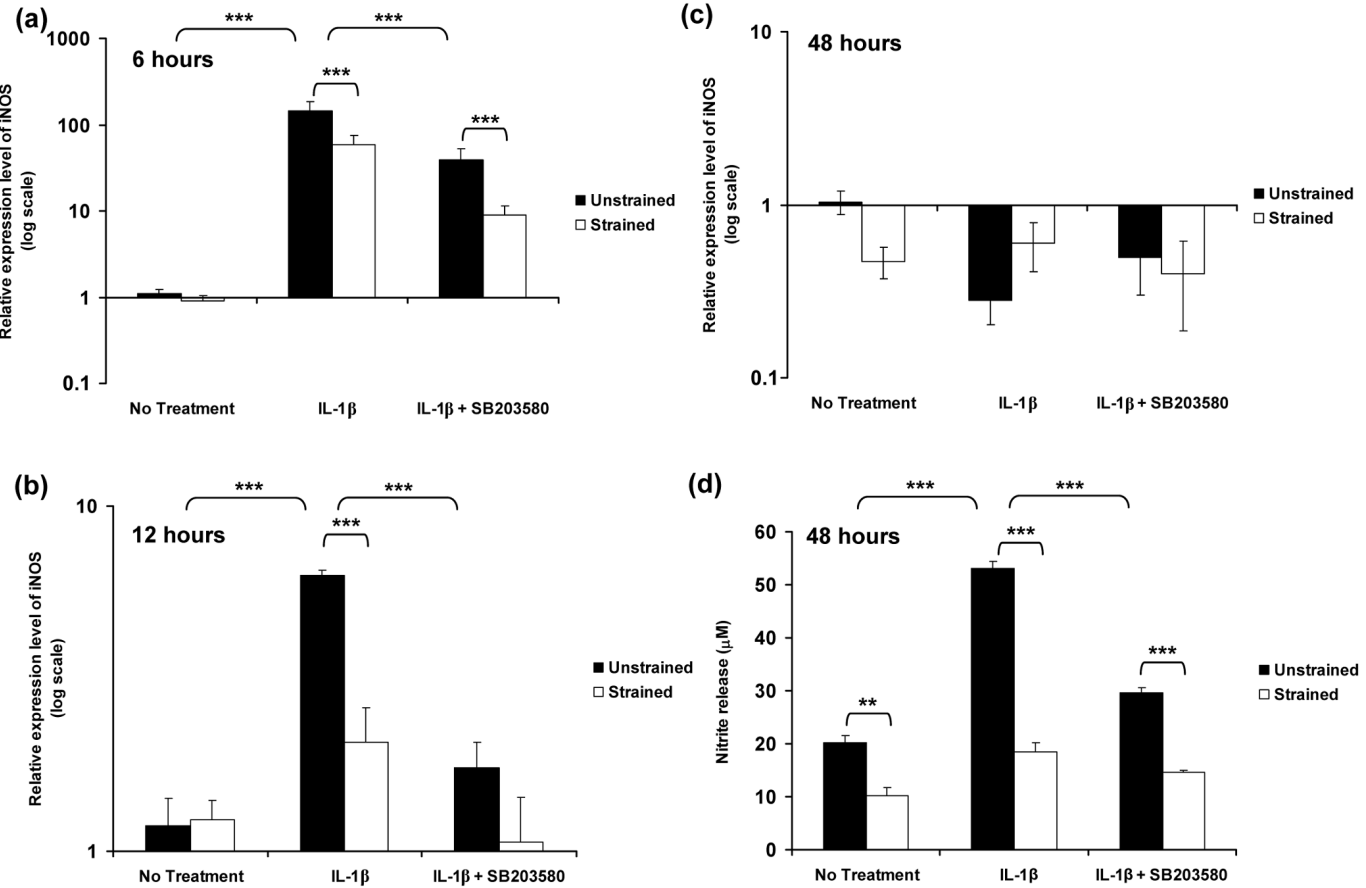
**Dynamic compression inhibits IL-1β induced iNOS expression and production of nitrite release**. Effects of 15% dynamic compressive strain on **(a,b,c) **iNOS expression and **(d) **nitrite production in unstrained and strained constructs cultured under no treatment conditions or with 10 ng/ml IL-1β and/or 10 μmol/l SB203580 for 6, 12 and 48 hours. The ratio of the relative expression level of iNOS was calibrated to the mean value for the unstrained (untreated) control and normalized to GAPDH. Ratios were expressed on a logarithmic scale (arbitrary units). Bars represent the mean and standard error of the mean of 16 to 18 replicates from four separate experiments. Two-way analysis of variance with *post hoc *Bonferroni-corrected *t*-tests was used to compare data: **P < 0.01 and ***P < 0.001. GAPDH, glyceraldehyde 3-phosphate dehydrogenase; iNOS, inducible isoforms of the nitric oxide synthase.

The relative expression levels of iNOS expression in unstrained constructs and constructs subjected to 15% dynamic compressive strain for 6, 12 and 48 hours are presented in Figure 4. The temporal profile of iNOS expression in response to IL-1β was similar to that in free-swelling culture, with a peak in expression at 6 hours (*P *< 0.001; Figure 4a). However, the magnitude of the peak expression induced by the cytokine was markedly greater than under free-swelling conditions, with approximately 145-fold increase in unstrained constructs (Figure 4a) as compared with sevenfold increase in free-swelling culture (Figure 4a). Stimulation by dynamic compression or SB203580 in isolation significantly reduced the IL-1β induced iNOS expression at both 6 and 12 hours (both *P *< 0.001; Figure 4a,b). However, co-stimulation with dynamic compression and the inhibitor produced an effect greater than each in isolation and resulted in iNOS expression returning to basal values (Figure 4a,b,c). iNOS induction at 6 and 12 hours closely correlated with the production of nitrite, which was maximal after 48 hours of incubation with the cytokine (*P *< 0.001; Figure 4d). The IL-1β induced nitrite release was inhibited by dynamic compression in the absence and presence of SB203580 (both *P *< 0.001; Figure 4d).

**Figure 5:**
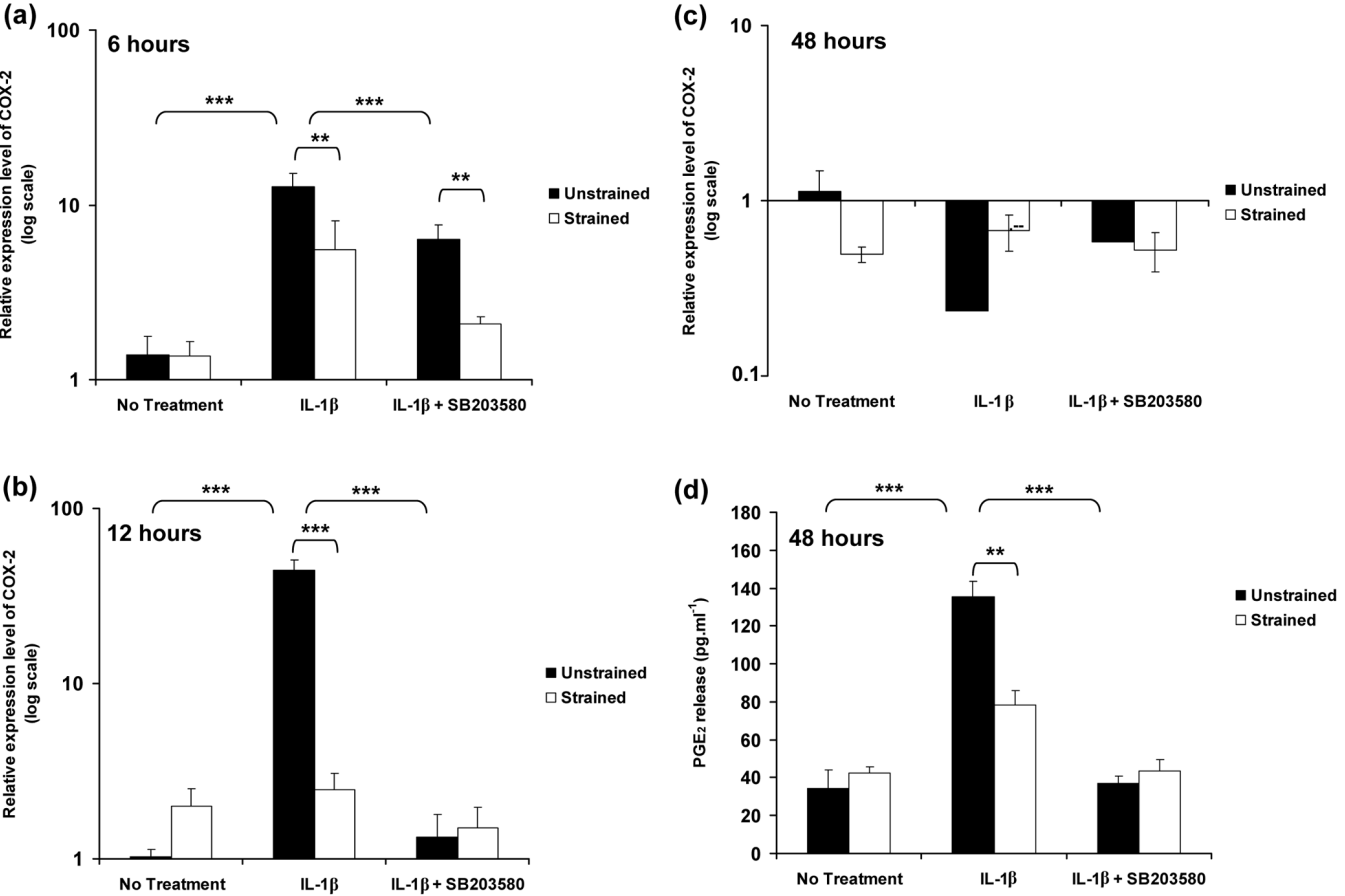
**Dynamic compression inhibits IL-1β induced COX-2 expression and production of PGE_2 _release**. Effects of 15% dynamic compressive strain on **(a,b,c) **COX-2 expression and **(d) **PGE_2 _production in unstrained and strained constructs cultured under no treatment conditions or with 10 ng/ml IL-1β and/or 10 μmol/l SB203580 for 6, 12 and 48 hours. The ratio of the relative expression level of COX-2 was calibrated to the mean value for the unstrained (untreated) control and normalized to GAPDH. Ratios were expressed on a logarithmic scale. Bars represent the mean and standard error of the mean of 16 to 18 replicates from four separate experiments. Two-way analysis of variance with *post hoc *Bonferroni-corrected *t*-tests was used to compare data: **P < 0.01 and ***P < 0.001. COX, cyclo-oxygenase; GAPDH, glyceraldehyde 3-phosphate dehydrogenase; PG, prostaglandin.

The temporal profile of COX-2 expression in unstrained and strained constructs, cultured for 6, 12 and 48 hours, is shown in Figure 5. The induction of COX-2 expression in response to IL-1β was similar to that in free-swelling culture, with peak expression at 12 hours (*P *< 0.001; Figure 5b). The relative magnitudes of the peak expression at 12 hours with the cytokine were 20-fold (Figure 2b) and 44-fold (Figure 5b) in free-swelling and unstrained constructs, respectively. The application of dynamic compression or presence of SB203580 reduced the IL-1β induced COX-2 expression at 6 hours (*P *< 0.01; Figure 5a). Stimulation by dynamic compression or SB203580 alone for 12 hours inhibited the cytokine-induced induction of COX-2 (both *P *< 0.001; Figure 5b). Co-stimulation with both dynamic compression and SB203580 had no further effect when compared with each in isolation at 12 hours (Figure 5b), with values returning to basal levels at 48 hours (Figure 5c). COX-2 expression correlated with PGE_2 _production with levels unaltered in unstrained and strained constructs cultured for 48 hours under no treatment conditions (Figure 5d). Dynamic compression (*P *< 0.01) or the presence of SB203580 (*P *< 0.001) alone abolished the IL-1β induced PGE_2 _release (Figure 5d). However, co-stimulation with both dynamic compression and SB203580 had no further effect compared with each in isolation (Figure 5d).

### Dynamic compression restored IL-1β induced inhibition of aggrecan expression

**Figure 6:**
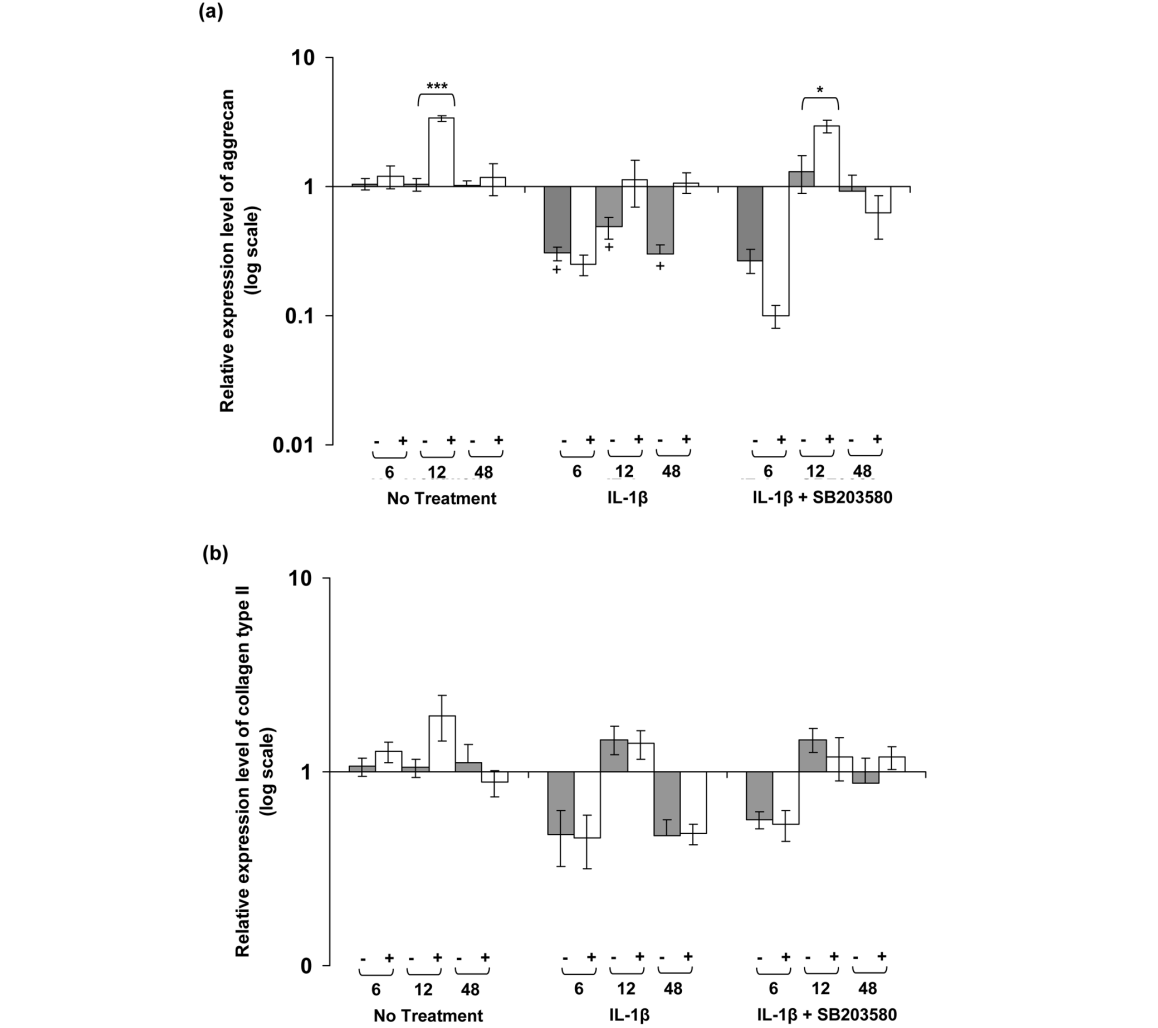
**IL-1β and dynamic compression influences aggrecan expression but not collagen type II**. Effects of 15% dynamic compressive strain on **(a) **aggrecan and **(b) **collagen type II expression in unstrained (-) and strained (+) constructs cultured under no treatment conditions or with 10 ng/ml IL-1β and/or 10 μmol/l SB203580 for 6, 12 and 48 hours. The ratio of the relative expression levels of the target gene was calibrated to the mean value for the unstrained (untreated) control and normalized to GAPDH. Ratios were expressed on a logarithmic scale (arbitrary units). Bars represent the mean and standard error of the mean of 10 replicates from three separate experiments. Two-way analysis of variance with *post hoc *Bonferroni-corrected *t*-tests was used to compare data: *P < 0.05 and ***P < 0.001 between unstrained and strained values; and ^+^*P *< 0.05 between untreated and IL-1β in unstrained constructs.

The expression levels for aggrecan and collagen type II in unstrained constructs and constructs subjected to dynamic compressive strain for 6, 12 and 48 hours are shown in Figure 6. In the absence of the cytokine, dynamic compression increased aggrecan expression to approximately threefold at 12 hours (*P *< 0.001; Figure 6a). IL-1β inhibited aggrecan expression in unstrained constructs at 6, 12 and 48 hours (all *P *< 0.05), and the levels were not influenced significantly by dynamic compression. However, co-stimulation with dynamic compression and SB203580 increased aggrecan expression at 12 hours (*P *< 0.05; Figure 6a). By contrast, the expression levels of collagen type II were not influenced by dynamic compression at 6, 12, or 48 hours in the presence or absence of the cytokine (Figure 6b). Moreover, the presence of the p38 MAPK inhibitor did not significantly influence collagen type II expression in unstrained or strained constructs.

## Discussion

IL-1β plays a pivotal role in both the pathogenesis of OA and as a potent mediator of catabolic processes in articular chondrocytes. The inflammatory process involves the excessive production of NO, PGs, matrix metalloproteinases, aggrecanases, and cytokines associated with the IL-1 and tumour necrosis factor families [5-9]. The net result is a tissue milieu with increased levels of inflammatory mediators that lead to cellular stress, chondrocyte apoptosis and loss of matrix tissue [5-9]. There is clinical evidence to support the beneficial effects of controlled moderate exercise in relieving adults with OA, in combination with chondroprotective agents [49,50]. However, the success of these treatments largely relies on the validation of the drugs and their pathophysiological interactions within the OA affected joint. Consequently, *in vitro *bioreactor systems have been developed as model systems to investigate the mechano-sensitive and cytokine-sensitive intracellular pathways. More specifically, a number of studies have shown that the application of controlled loading regimens at physiological magnitude prevents the harmful effects of IL-1β by blocking the release of NO, PGE_2 _and matrix metalloproteinases, and restoring extracellular matrix production [10-18,38,39,45,46]. Consequently, the tissue maintains a balance in matrix turnover and regulates cytokine-induced pathways during articular cartilage loading.

In the present study, we utilized real-time quantitative PCR assays coupled with a novel fluorescent probe known as the molecular beacon to detect the catabolic (iNOS and COX-2) and anabolic (aggrecan and collagen type II) genes utilizing the chondrocyte/agarose model in conjunction with a well characterized bioreactor system [40,41]. The data provide striking evidence that dynamic compression antagonizes the IL-1β induced expression of iNOS and COX-2 levels and production of NO and PGE_2 _release. Additionally, we provide evidence to support restoration of aggrecan levels by both types of stimuli. These data support our previous studies that show that dynamic compression counteracts the IL-1β induced production of NO and PGE_2 _release by full-depth chondrocytes and superficial zone chondrocytes, and restores cell proliferation and proteoglycan synthesis [16,17].

The free-swelling studies were undertaken to determine how the cytokine influenced gene expression levels with time, in the absence of the bioreactor system. IL-1β activated the phosphorylation of p38 MAPK and was inhibited by the p38 MAPK inhibitor (Figure 1). The cytokine additionally induced a transient increase in the expression levels of both iNOS and COX-2 (Figure 2). COX-2 expression appeared to be more sensitive to the cytokine and to the presence of SB203580 (Figure 2b), suggesting that COX-2 activation may occur independently of NO and primarily involves a p38 MAPK dependent pathway. We observed a similar effect with the release of NO (Figure 3a) and PGE_2 _production (Figure 3b) in response to IL-1β, in which SB203580 had a more potent effect on PGE_2 _release than on NO production. It could be argued that the p38 MAPK inhibitor may have nonspecific effects. Previous studies have shown inhibition of NO release by SB203580 at a concentration of 1 and 10 μmol/l in IL-1β treated chondrocytes [20-22,24,51]. However, at concentrations at 10 μmol/l or greater, SB203580 was demonstrated to inhibit JNK in chondrocyte monolayers, suggesting less specificity at a higher dose [52]. Accordingly, iNOS induction and NO release may therefore involve activation of multiple MAPK pathways, in particular JNK. A number of previous studies have reported differential mechanisms through which IL-1β upregulates iNOS and COX-2 expression that involve activating transcription factor-2, NF-κB, IL-6, cAMP responsive element binding protein-1, or activator protein-2 transcription factors and activation of all members of the MAPKs [20,23,53]. Nevertheless, NF-κB appears to be the primary transcription factor that influences iNOS expression and NO production, and has been shown to strongly inhibit PGE_2 _synthesis in OA affected cartilage [52]. Our studies utilizing human chondrocyte/agarose constructs cultured with selective inhibitors of iNOS (1400W) and COX-2 (NS-398), also suggest that NO could have a negative influence on PGE_2 _production [18]. However, whether NO induces or suppresses PGE_2 _release in the presence of IL-1β remains controversial [9,18,54].

In separate experiments, constructs were subjected to dynamic compression or remained unstrained within a bioreactor system. The present data shows that unstrained culture within the bioreactor system induced a significant enhancement in the levels of NO release in the presence and absence of IL-1β (Figure 4d), when compared with free-swelling culture conditions (Figure 2a), whereas PGE_2 _release (Figures 2b and 5d) was largely unaffected, a phenomenon that concurs with our previous studies [16,17]. A similar effect was noted for iNOS induction in response to IL-1β, with a sevenfold increase in free-swelling conditions (Figure 2a), as compared with a 145-fold increase in unstrained constructs cultured within the bioreactor without the application of dynamic compression (Figure 4a). In accordance with the PGE_2 _release data, the fold increase in expression of COX-2 was broadly similar under both culture conditions (Figures 2b and 5). There are marked differences in the diffusional constraints in unstrained constructs in the bioreactor, involving contact of the upper surface with a fluid impermeable loading pin. Accordingly, the mass transport of oxygen into the construct may be impaired, leading to increased hypoxia in the bioreactor system, compared with constructs cultured under free-swelling conditions. A number of recent studies support the hypothesis that oxygen tension can influence the production of inflammatory mediators in cartilage. For example, hypoxia dramatically increased iNOS expression and NO production by bovine chondrocytes in response to IL-1β, whereas COX-2 expression was not strongly influenced by oxygen tension [44,55,56].

Co-stimulation with dynamic compression and inhibitor for 6 hours completely abolished iNOS induction in response to IL-1β (Figure 4a). Interestingly, neither dynamic compression or the inhibitor alone were able to abolish the IL-1β induced iNOS expression. The effect was more striking for COX-2 with levels returning to basal values after application of dynamic compression or the p38 MAPK inhibitor alone for 12 hours (Figure 5b). The data suggest interactions between mechano-sensitive and p38 MAPK dependent pathways in mediating the inhibitory effect of dynamic compression on iNOS and COX-2 expression. The importance of these findings is supported by recent studies utilizing monolayer chondrocytes, which showed sustained levels of iNOS and COX-2 expression in response to IL-1β for up to 24 hours, with a dramatic reduction by cyclic tensile strain for 8 hours over a 24-hour loading regimen [15]. Additionally, the mechanism was shown to involve NF-κB activation, which could act downstream of p38 MAPK [13]. Nonetheless, static or intermittent compression of different magnitudes applied to cartilage explants increased NO and PGE_2 _production [28,29]. These differences may potentially be attributed to both the nature of the mechanical stimulus and the level of oxygen tension, as indicated in recent studies [56-58].

We initially examined the involvement of the p38 MAPK because this kinase was influenced by either IL-1β or mechanical loading [24,52]. We demonstrated activation of the p38 MAPK following 20 minutes of stimulation with the cytokine (Figure 1). However, a major limitation of the bioreactor system is the time taken to retrieve constructs at the end of an experiment (up to 10 minutes). This causes difficulties associated with analyzing transient phosphorylation events, because alterations in activation state may occur after the end of the mechanical loading regimen. Accordingly, the time courses of phosphorylation events in response to both IL-1β and dynamic compression are not presented here, but work is actively ongoing. In addition, we feel that the regulation of the IL-1β pathways may be through many factors, and dynamic compression may actually target a global mechanism involving JNK or NF-κB, as indicated in previous studies [13,51,52].

In addition to the regulation of iNOS and COX-2 by dynamic compression, co-stimulation with the inhibitor was important in restoring the cytokine-induced inhibition of aggrecan expression. This is in contrast to collagen type II, in which no response to IL-1β or dynamic compression was observed. It is highly likely that matrix gene expression is controlled by transient factors, and could involve rapid activation of multiple transcription factors that are influenced by a combination of mechanical loading and IL-1β [19,22,31,32]. Consequently, the multiple events activated in a time-dependent manner will influence the rate of expression and assembly of the extracellular matrix molecules, causing flare-ups in gene expression levels. This phenomenon may start as early as 1 hour after mechanical stimulation, as reported with aggrecan and collagen type II expression [37,59].

Overall, these observations indicate a hypothetical mechanism for the activation of iNOS by IL-1β that are, respectively, dependent on p38 MAPK signalling and could involve activation of other MAPKs. In free-swelling conditions the p38 independent pathway is predominantly activated, as indicated by the partial effect of the p38 inhibitor on iNOS expression at later time points. However, at early time points the p38 inhibitor blocked activation of the p38 MAPK. Incorporation of the construct into the bioreactor system, with its inherent diffusional constraints, dramatically enhances iNOS induction, potentially via preferential activation of the p38 MAPK dependent pathway and possibly through a hypoxia-driven mechanism. Accordingly, the inhibitor has a greater effect on iNOS expression under these conditions. Dynamic compression substantially reduces iNOS expression and co-stimulation with both dynamic compression and the p38 MAPK inhibitor abolished iNOS expression when compared with each in isolation, suggesting that dynamic compression targets the p38 MAPK dependent pathway. By contrast, COX-2 activation by IL-1β appears to be primarily mediated by a p38 MAPK dependent pathway that is largely unaffected by transfer to the bioreactor system but is highly susceptible to the p38 MAPK inhibitor or dynamic compression. In addition to the regulation of iNOS and COX-2 expression by dynamic compression, co-stimulation with the inhibitor was important in restoring the cytokine-induced inhibition of aggrecan expression. This is in contrast to collagen type II, in which we observed no response. It is probable that matrix gene expression is controlled by transient factors and could involve rapid activation of multiple MAPKs and transcription factors. Ultimately, elucidation of the intracellular pathways will enable the identification of appropriate pharmacological agents and provide a clinical rationale for promoting the benefits of controlled physical activity to manage and treat OA.

## Conclusion

It is important to define the mechanistic pathways induced by physiologically relevant mechanical signals in the presence of proinflammatory mediators, because this information will provide key parameters for the safe application of pharmacological therapies, in conjunction with biophysical treatments for OA.

## Abbreviations

COX = cyclo-oxygenase; C_t _= cycle threshold; GAPDH = glyceraldehyde 3-phosphate dehydrogenase; IL = interleukin; iNOS = inducible isoforms of the nitric oxide synthase; JNK = c-Jun amino-terminal kinase; MAPK = mitogen-activated protein kinase; NF-κB = nuclear factor-κB; NO = nitric oxide; OA = osteoarthritis; PCR = polymerase chain reaction; PG = prostaglandin.

## Competing interests

The authors declare that they have no competing interests.

## Authors' contributions

TC supervised SA and OOA, who performed cell culture experiments and analysis by real-time PCR. DS and JB participated in Western blot analysis and TC carried out the biochemical assays, performed the statistical analysis and drafted the manuscript. DS, DB and DL participated in its design and coordination and helped to draft the manuscript. All authors read and approved the final manuscript.
